# Acute Resveratrol Consumption Improves Neurovascular Coupling Capacity in Adults with Type 2 Diabetes Mellitus

**DOI:** 10.3390/nu8070425

**Published:** 2016-07-12

**Authors:** Rachel H.X. Wong, Daniel Raederstorff, Peter R.C. Howe

**Affiliations:** 1 Clinical Nutrition Research Centre, School of Biomedical Sciences and Pharmacy, University of Newcastle, Callaghan, NSW 2308, Australia; rachel.wong@newcastle.edu.au; 2 DSM Nutritional Products Ltd., P.O. Box 2676, 4002 Basel, Switzerland; daniel.raederstorff@dsm.com

**Keywords:** resveratrol, type 2 diabetes, cerebral perfusion, cognitive function, neurovascular coupling, transcranial Doppler ultrasound, multi-tasking, randomized controlled trial

## Abstract

Background: Poor cerebral perfusion may contribute to cognitive impairment in type 2 diabetes mellitus (T2DM). We conducted a randomized controlled trial to test the hypothesis that resveratrol can enhance cerebral vasodilator function and thereby alleviate the cognitive deficits in T2DM. We have already reported that acute resveratrol consumption improved cerebrovascular responsiveness (CVR) to hypercapnia. We now report the effects of resveratrol on neurovascular coupling capacity (CVR to cognitive stimuli), cognitive performance and correlations with plasma resveratrol concentrations. Methods: Thirty-six T2DM adults aged 40–80 years were randomized to consume single doses of resveratrol (0, 75, 150 and 300 mg) at weekly intervals. Transcranial Doppler ultrasound was used to monitor changes in blood flow velocity (BFV) during a cognitive test battery. The battery consisted of dual-tasking (finger tapping with both Trail Making task and Serial Subtraction 3 task) and a computerized multi-tasking test that required attending to four tasks simultaneously. CVR to cognitive tasks was calculated as the per cent increase in BFV from pre-test basal to peak mean blood flow velocity and also as the area under the curve for BFV. Results: Compared to placebo, 75 mg resveratrol significantly improved neurovascular coupling capacity, which correlated with plasma total resveratrol levels. Enhanced performance on the multi-tasking test battery was also evident following 75 mg and 300 mg of resveratrol. Conclusion: a single 75 mg dose of resveratrol was able to improve neurovascular coupling and cognitive performance in T2DM. Evaluation of benefits of chronic resveratrol supplementation is now warranted.

## 1. Introduction

Both age and diabetes status contribute to cognitive impairment. One of the proposed underlying mechanisms for this deficit is poor cerebral perfusion, which is likely due to the progressive microvascular dysfunction associated with the formation of advanced glycation end products (AGEs) in type 2 diabetes mellitus (T2DM) [1]. The association between cerebral perfusion and severity of cognitive impairment is well established [2]. Undoubtedly, adequate blood flow is vital for brain function. When neurons fire, the signalling for more blood flow is communicated to the endothelium to release nitric oxide (NO), resulting in dilatation of local arterioles, which is reflected in increased blood flow in the larger arteries supplying the brain region. This response is termed neurovascular coupling, where changes in cerebral blood flow are tightly coupled to specific neuronal events [3]. As such, the health of the endothelium is crucial for facilitating the rapid supply of blood on demand and there are compelling reasons to believe that dysfunction between neurons and blood vessels can impair each other. In T2DM, AGEs can trigger overexcitation of microglia in the CNS, causing neuronal damage. Together with age-related cell senesce, AGEs can also increase the influx of inflammatory cytokines and proliferation of smooth muscle cells, resulting in arterial stiffness and impairment of NO-mediated vasodilatation [4,5]. Short-term deprivation of blood during neuronal activation may result in poor cognitive performance. Moreover, chronic hypoperfusion could result in a more extensive irreversible damage to the network of brain cells, making it difficult to achieve optimal cognitive function.

Transcranial Doppler (TCD) ultrasound monitoring of blood flow velocity (BFV) in the middle cerebral artery (MCA) has shown that the cerebral hypoperfusion in T2DM adults is attributable to global intracranial stenosis. Under basal conditions, this is characterized by reduced cerebral blood flow velocity and increased pulsatility index (a measure of intracranial vessel stiffness) [6,7]. In response to a physiological stimulus (viz. hypercapnia), T2DM adults have impaired cerebral vasodilatation [8]. Impaired cerebrovascular function is predictive of cerebrovascular events [9]. Neuroimaging further reveals deficits in specific hypercapnia-induced vasodilatory capacity in the parietal-occipital and cortical regions of T2DM adults compared with non-T2DM [7]. The lack of uniformity in cerebrovascular dysfunction may explain the particular profile of cognitive deficit that is implicated in T2DM [10]. Indeed, neurovascular coupling is compromised in T2DM when assessed by BOLD signal during stimulation of visual cortex, suggesting that performance on a mental task may be compromised should the particular brain region be under-perfused [11]. Hence, evaluating local brain region blood flow demands during mental activation rather than global vasodilatory responses such cerebrovascular responsiveness (CVR) to hypercapnia may provide a better insight to cognitive performance, where brain region activation is task dependent.

In a cross-sectional observation [8], we found that T2DM adults have persistent microvascular dysfunction in the cerebral vessels and sub-clinical cognitive decline compared with their age-gender matched non-T2DM counterparts, despite proper metabolic control with current management strategies (i.e., oral hypoglycaemic medications). Importantly, cerebral perfusion during mental activation, measured by TCD, is reduced [8]. Executive and attentional function deficits are reported in the T2DM population where daily activities such as planning, decision-making, ability to modify goal-oriented behaviour to a novel situation and doing two things simultaneously are compromised [10,12]. Notably, these activities rely heavily on attentional resources and attention is a known finite resource [13]. On this basis, T2DM adults may face increasing cognitive demand of doing two things at the same time (such as walking and talking) or exhibit difficulties switching attention between tasks [14,15]. As such it has been proposed that assessing cognitive capacity in T2DM adults with normal cognition using complex tasks that require sharing of brain resources (i.e., dual-tasking) may be better suited for distinguishing performance or evaluating the effectiveness of intervention in a high-functioning cohort [10]. There are currently no studies reporting on the magnitude of cerebral perfusion during dual-type tasks in T2DM.

Evidence that certain *vasoactive* nutrients can boost cerebral blood flow and improve CVR to various stimuli is mounting [16,17,18]. Resveratrol, a vasoactive ingredient present in red grapes, has shown to be most promising. We have previously shown that resveratrol can dose-dependently enhance vasodilatation acutely in overweight hypertensive adults, as measured by flow-mediated dilatation (FMD) of the brachial artery [19]. Furthermore, daily supplementation with 75 mg of resveratrol for six weeks resulted in a sustained enhancement of FMD in healthy older adults with age-related cognitive decline and endothelial dysfunction. A trend for improvement in an executive function task was also observed [20]. This led to our hypothesis that the enhancement of vasodilator function may improve cognitive performance [21]. However, improvements in vasodilator responsiveness may differ between the systemic and cerebral circulations, depending on the nature of the vasoactive mediator [17]. Therefore, it is important to identify the optimal resveratrol dose for enhancing cerebral perfusion, which may in turn influence cognitive performance in this population.

Recently, we reported that 75 mg and 300 mg of resveratrol were efficacious compared with 150 mg for improving CVR to hypercapnia in older adults with well-controlled T2DM [18]. In the same study, we also aimed to evaluate the neurovascular coupling capacity and its influence on cognitive performance during dual and multi-task conditions following acute resveratrol consumption and the relationship to plasma total resveratrol levels. We now report these outcomes.

## 2. Materials and Methods

An acute randomised, double-blind, placebo-controlled dietary intervention was undertaken at the University of Newcastle’s Clinical Nutrition Research Centre. The study was approved by the University of Newcastle Human Research Ethics Committee, registered with the Australia and New Zealand Clinical Trials Registry (ACTRN12614000891628) and conducted according to the International Conference on Harmonisation guidelines for Good Clinical Practices. Adults aged 40 to 80 years with a diagnosis of T2DM were recruited from the Hunter region in Australia via radio and newspaper announcements. All participants provided written, informed consent prior to enrolment.

The screening protocol and method of randomization and masking for this study have been previously reported [18]. The four doses of resveratrol used in this study were 0, 75, 150 and 300 mg of synthetic trans-resveratrol (>99% purity, DSM Nutritional Products Ltd., Basel, Switzerland). 

### 2.1. Schedule of Assessments

Participants arrived at the research centre following a 2 h fast (no food/beverage, medication or supplement, except water). They were given a standard meal containing apple juice (with no added sugar) and a low-glycaemic index (GI = 54) muesli bar to consume within 10 mins along with their assigned dose of resveratrol to be taken with water. Following a 75 min wait, their CVR in the MCA to a battery of cognitive tasks using TCD commenced (see [22] for a detailed description of this technique). Prior to the start of each cognitive test, a 30 s basal blood flow velocity was determined. CVR to each cognitive test using TCD was recorded. The cognitive test battery required participants to perform two or more tasks at the same time, thus requiring divided attention, which is closely matched to everyday activities. The rationale for the choice of cognitive tests has been previously reviewed [10]. Blood for analysis of plasma total resveratrol was obtained pre-supplementation and at the conclusion of the cognitive test battery (~120 min post-supplementation).

#### 2.1.1. Computerised Multi-Tasking Test Battery

A computerized multi-tasking test battery (Purple Research Framework, UK) was then administered. Participants were presented with four tasks simultaneously on a computer screen to which they were instructed to attend and respond quickly to each task equally for 5 min. In one task window, participants scanned through a 4 x 4 matrix of single numbers and select the highest digits. Upon successful selection, a fresh matrix of numbers was presented. This task assesses visual scanning and attention. In another task of working memory, the participant had to memorise five randomly generated letters within 10 s. After the string of letters disappeared, a randomly generated letter was presented; the participants clicked “yes” if the letter was from the previously presented letters. In another task window, six rectangular bars rose at different rates. As soon as one of the red bars reached the top, a “warning sign” flashed. The participant then attended to the task by selecting the bars in numerical sequence. The task was repeated upon successful completion. This task served as a distractor to the other tasks. Points were deducted if the participant failed to attend to the task requirement. The fourth task window consisted of four coloured blocks (red, green, blue and yellow) and the name of a colour printed in incongruent ink. The participants were asked to click on the colour block that corresponded with the colour of the text, not the word. This Stroop colour–word task is an assessment of executive function.

Points were awarded for correctly responding to tasks and were negatively scored for mistakes made or failure to attend to a task within a randomly allocated time. Each task score was converted to percentage accuracy (i.e., the number of correct responses/a fixed number that was standardized for every dose/visit, multiplied by 100). Performance on the computerized multi-tasking test battery was determined by averaging the percentage accuracy for the four tasks. This average was also divided by the average time taken to respond to each task on the multi-tasking test battery, giving a performance index. A higher value meant better performance.

#### 2.1.2. Dual Tasking Test Battery

To increase the cognitive load and task complexity, participants were required to perform the finger tapping task on the keyboard while completing the Trial Making Task (TMT) and the Serial Subtraction 3 (SS3) task verbally each for 1 min. The participants first performed the tapping task with the index finger of the dominant hand and non-dominant hand as fast as they could for 1 min each. The tapping component served as a distractor and was used to determine the cognitive contributions arising from the dual-tasking component of the test battery. In the TMT, they were asked to say aloud alternating numbers and letters in ascending order (i.e., 1-A-2-B-3-C-4-D, etc.). The TMT assesses mental switching and the ability to ignore distractions and irrelevant information [23]. In the SS3, participants subtracted a series of threes from a random starting number. A different three digit starting number was given for each of the four intervention visits to minimize practice effects. Percentage accuracy in performance on the TMT and the SS3 was calculated by dividing the number of correct responses by a fixed number (e.g., 200 was the limit for SS3). Overall cognitive performance was taken as an average of the computerized multi-tasking test and dual-tasking tests.

### 2.2. Outcomes

The primary outcome of this intervention trial, as documented in the clinical trial registration, was the acute effect of resveratrol consumption on the cerebrovascular response to a physiological stimulus, viz. hypercapnia, which has been reported previously [18]. We had sufficient information from previous trials to estimate statistical power for this outcome. We now report the effect of resveratrol consumption on neurovascular coupling capacity, a new outcome of equivalent importance, on plasma resveratrol levels (listed as a secondary outcome) and on measures of cognitive performance, which we regard as exploratory outcomes, as we had no previous indication that they would be modified acutely by resveratrol.

#### 2.2.1. Analysis of Neurovascular Coupling Capacity

Increases of BFV in the MCA in response to cognitive stimuli are proxy measures of neurovascular coupling capacity; they reflect the extent of vasodilator capacity in downstream vascular beds during neuronal demand [24]. These CVR to cognitive stimuli are calculated using two methods. The first is the peak increase in mean BFV, expressed as a percentage of the mean BFV recorded under basal pre-test conditions. The second is the area under the curve (AUC), calculated by the trapezoidal method, for the change in mean BFV during the cognitive tasks, which had a defined duration. AUC during the tapping and oral TMT and tapping and oral SS3 were determined by subtracting the AUC during the tapping task only, thus compensating for any rise in BFV from a motor task that had little or no cognitive contribution. The overall CVR to cognitive tasks was determined as an average of the CVR to each task in the cognitive test battery.

#### 2.2.2. Plasma Resveratrol Analysis

Total trans-resveratrol (sum of aglycone and conjugates) was measured in plasma derived from venous blood samples obtained before and after resveratrol consumption. Briefly, labelled internal standard was added to an aliquot of plasma and a β-glucuronidase digestion was performed before the liquid-liquid extraction. After centrifugation an aliquot of the organic phase was evaporated to dryness, re-dissolved in injection solvent and analysed on LC-MS/MS system on a C18 column. A log transformation was applied to the total trans-resveratrol levels for correlational analysis.

### 2.3. Statistical Analysis

A paired t-test was used to determine differences in bilateral MCA responses. If no statistical difference existed, both left and right MCA responses were averaged and used for analysis. Repeated measures ANOVA (IBM^®^ SPSS^®^ Version 21, New York, NY, USA) were performed on the CVR to cognitive stimuli, overall cognitive performance and plasma resveratrol concentrations to determine the significance of differences between each dose of resveratrol. Linear regression was also used to determine whether changes in log of plasma resveratrol concentrations were related to changes in CVR to cognitive stimuli and overall cognitive performance. False discovery rate was applied to correct for multiple comparisons (significance level was set at *P* = 0.038). All results are presented as mean ± SEM.

## 3. Results

Thirty-eight participants with T2DM met the selection criteria and were enrolled in the study. Two withdrew their consent to participate before the first intervention visit; 36 participants (26 men and 10 postmenopausal women) completed the study. Their baseline/screening characteristics were detailed in our previous report [18]. They had an average age of 68.5 years and average BMI of 30.3 kg/m^2^. Eight were using diet and exercise alone to manage their T2DM, while the remainder took oral hypoglycaemic agents; HbA1c averaged 6.7%, indicating that their diabetes was well controlled. Their scores on the Australian version of the Modified Mini Mental State Examination (3MS) were within normal limits.

### 3.1. Neurovascular Coupling Capacity

**Figure 1:**
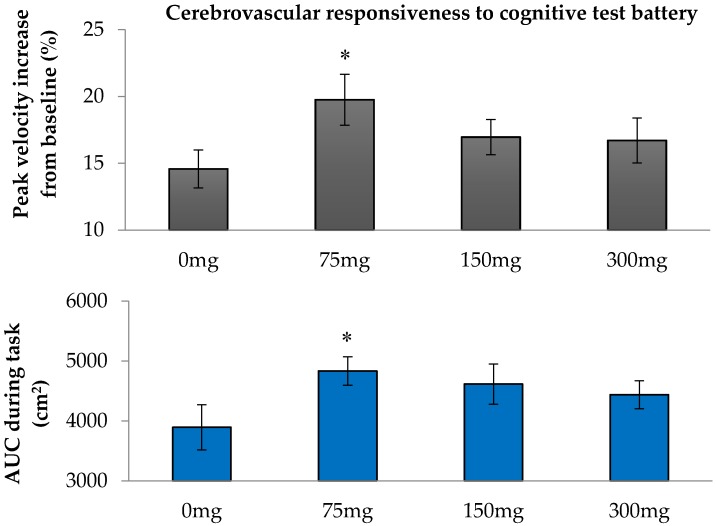
Overall cerebrovascular responsiveness to dual and multi-tasking test batteries for placebo and three resveratrol doses: (**Top**) the percentage increase in mean blood flow velocity (BFV) from basal velocity; and (**bottom**) area under the curve (AUC) of the BFV responses to tests. Data are mean ± SEM. * *P* < 0.038 compared to placebo.

The overall CVR to the dual and multi-tasking test batteries recorded at each visit is shown in Figure 1. The responses to cognitive testing did not differ statistically between left and right MCA and were therefore averaged. Compared with placebo (0 mg), the 75 mg dose of resveratrol elicited a 35% greater increase in BFV during the dual and multi-tasking test batteries (*P* = 0.019). This improvement was confirmed in the AUC analysis of BFV responses during the task (25% increase, *P* = 0.025), which correlated with the within-individual per cent increases in BFV to the test battery (*r* = 0.213, *P* = 0.017), indicating agreement between the two methods of analysis.

### 3.2. Task Performance

Table 1 details the overall accuracy of performance to the dual and multi-tasking test battery and the accuracy for individual tasks. Compared with placebo, there were no significant treatment changes to each of the cognitive tests and the overall cognitive performance. However, there was a trend towards an improvement on the tapping + SS3 task following resveratrol consumption compared with placebo (75 mg: 3.1% ± 1.6%, *P* = 0.058; 150 mg: 1.0% ± 1.4%, *P* = 0.498; 300 mg: 2.7 ± 1.4, *P* = 0.055). Despite similar scores on the accuracy of performance in the computerized multitasking test battery, the average time taken to respond to each task in the multi-tasking battery was lower following a single dose of resveratrol. Hence, the performance index (accuracy/time) on the computerized multi-tasking test battery was significantly enhanced by both 75 mg and 300 mg doses of resveratrol compared to placebo (75 mg: 0.50 ± 0.09, *P* < 0.001; 150 mg: 0.00 ± 0.03, *P* = 0.975; 300 mg: 0.55 ± 0.08, *P* <0.001).

### 3.3. Plasma Resveratrol Concentrations

**Figure 2:**
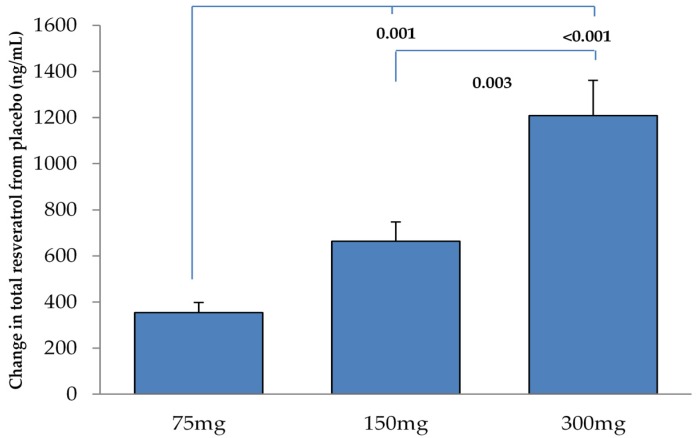
Changes in total plasma resveratrol concentrations with each dose of resveratrol compared to placebo. Data are mean ± SEM.

No trans-resveratrol was detected in the pre-supplement blood samples across all doses, confirming no carryover effects from the previous treatment dose. Plasma concentrations of total trans-resveratrol following 0 (placebo), 75, 150 and 300 mg were 0 ng/mL, 351 ± 43 ng/mL, 688 ± 84 ng/mL and 1241 ± 147 ng/mL, respectively. As expected, there was a significant dose–response relationship (*r* = 0.661, *P* < 0.001). Plasma resveratrol concentrations were also significantly different between resveratrol doses (See Figure 2).

### 3.4. Correlations between Log Plasma Total Resveratrol Levels and Outcome Measures

We have previously reported the effect of resveratrol doses on CVR to hypercapnia in this study [18]. Changes in log plasma total resveratrol concentration correlated with changes in CVR to hypercapnia in the MCA (*r* = 0.296, *P* = 0.001) but not with CVR to hypercapnia in the posterior cerebral artery (*r* = 0.210; *P* = 0.081).

Changes in log plasma total resveratrol levels correlated with CVR (AUC) to the cognitive test battery (AUC analysis: *r* = 0.223, *P* = 0.011; per cent increase in mean blood flow velocity analysis: *r* = 0.114, *P* = 0.200) but not with overall cognitive performance (*r* = 0.026, *P* = 0.759).

## 4. Discussion

One of the aims of this study was to determine whether a single dose of resveratrol could enhance neurovascular coupling capacity in adults with T2DM. We report that the lowest resveratrol dose of 75 mg used in the study was optimal for enhancing CVR to selected cognitive stimuli, which also correlated with the increase in plasma resveratrol concentration. This is in line with our previous finding that the 75 mg and 300 mg resveratrol doses were able to enhance CVR to hypercapnia in this population [18]. We also investigated whether the increase in neurovascular coupling capacity would influence cognitive performance acutely. While there was no significant change in overall cognitive performance, a performance index on the computerized multi-tasking test battery (ratio of accuracy to response time) was improved by both 75 mg and 300 mg of resveratrol.

Kennedy et al. previously showed a dose dependent increase in cerebral blood flow during task performance in healthy young adults following 250 mg and 500 mg of resveratrol [16]. Our findings indicated that the 75 mg resveratrol dose was more efficacious in enhancing cerebral perfusion during mental task performance of T2DM adults than the highest dose of 300 mg used. In our study, the test battery commenced 75 mins after consumption of resveratrol and lasted until 120 min. In contrast, the cognitive test battery in Kennedy’s study commenced 45 min post supplementation and lasted for 40 min. While their 500 mg resveratrol dose gave significantly greater responses than placebo at each 5 min epoch, the mean increase of blood flow appeared to be declining towards the end of the test battery. On the other hand, the increase in cerebral blood flow following their 250 mg dose did not exhibit this decline and, in fact, at epoch 73–76 min, the increase of blood flow from placebo was significant. The total plasma resveratrol concentration following the 250 mg resveratrol dose remained elevated between 90 and 120 min post resveratrol consumption. In contrast, total plasma resveratrol concentrations with the 500 mg dose showed a decline during this period [16]. Taken together, these observations suggest that a lower dose of resveratrol may exert longer lasting effects than higher doses. Indeed, recent studies suggest that exposure to low doses of resveratrol trigger mild cellular stress responses, which in turn upregulate multiple stress resistance proteins to protect the neuron against injury. In the case of resveratrol, this hormesis-based mechanism of action is triggered at low doses through the activation of SIRT1 to activate a FOXO3 pathway to induce the expression of brain-derived neurotrophic factor, a protein responsible for synaptogenesis and neurogenesis [25]. At high doses, resveratrol is pro-apoptotic and causes DNA strand breakage due to the presence of free radicals [26].

One of the strengths of this study is the correlation between CVR to the cognitive test battery and plasma total resveratrol levels. While there is evidence in rat models of cerebral ischaemic injury that resveratrol in the form of a glucuronide conjugate can cross the blood-brain barrier to be taken up by brain tissue, this has yet to be tested in humans [27]. Nonetheless, the vasodilator effect of resveratrol following a single dose is most likely to be through rapid activation of oestrogen receptor (ER) signalling in endothelial cells. Specifically, resveratrol binds to and increases the transcriptional activity of ER-α and ER-β to activate MAPK pathways to increase endothelial NO synthase. This in turn increases NO bioavailability and vasodilatation at the smooth muscle cell, thereby facilitating cerebral perfusion [28]. Both ER-α and ER-β receptors are expressed throughout the brain tissues with the highest concentrations present in the forebrain, hippocampus and amygdala, which governs high level cognition, learning, memory and mood, respectively [29]. Therefore, binding of resveratrol to ER in the brain may be a plausible means of augmenting neurovascular coupling seen in this study.

Both our study and that of Kennedy et al. [16] saw no significant change in cognitive performance following acute resveratrol consumption. While acute enhancement of cognitive performance in a healthy young adult population was not anticipated, the T2DM adults in our study showed marginal improvements in the tapping and oral SS3 task and a better performance index with the computerized multi-tasking test battery following 75 mg and 300 mg of resveratrol. This acute observation of a potential benefit warrants future evaluation in a chronic supplementation trial that utilises complex cognitive tasks, similar to the dual tasks and multi-tasking test battery used in this study. It is likely that the increased cerebral perfusion following resveratrol consumption contributed to this cognitive benefit. However, plasma resveratrol concentrations did not correlate with overall cognitive performances in this study. Intrinsic factors such as mood and level of motivation may have influenced individual task performance at each visit regardless of the effect of resveratrol treatment on cerebral perfusion. Moreover, only three cognitive tests were administered assessing limited cognitive domains and were therefore not representative of global cognitive function. The chronic evaluation of resveratrol in T2DM now warrants further investigation. We anticipate that regular consumption of resveratrol with 75 mg of resveratrol will not only enhance cerebrovascular function through both genomic (viz. SIRT1) and non-genomic pathways (i.e., ER signalling), which will in turn improve cognitive function, but will also counteract insulin resistance in T2DM, as previously discussed in our first report of this study [18].

## 5. Conclusions

Acute consumption of a single 75 mg dose of resveratrol by adults with T2DM was optimal for enhancing their CVR to selected cognitive stimuli, which also correlated with resultant increases in their plasma resveratrol concentration. Although acute treatment was not expected to enhance performance in the dual and multi-tasking test battery, we observed an improvement of performance index in the computerised multi-tasking test battery following both 75 mg and 300 mg of resveratrol. This study indicates the suitability of a low dose of resveratrol for evaluating chronic effects on cerebrovascular function and cognitive function in this population. 

## Figures and Tables

**Table 1:** Overall cognitive performance is calculated as the average percentage accuracy from dual tasking and computerized multi-tasking test battery. The performance index to the computerized multi-tasking test battery is determined by the ratio of the accuracy and response time taken for each task in the multi-tasking battery, where a higher value equates to better performance. Data are shown as mean ± SEM. * *P* < 0.001 compared to placebo.

Dose	Overall Cognitive Performance	Tapping + Oral TMT	Tapping + SS3	Computerised Multi-Tasking Test Battery	Average Response Time for Each Multi-Tasking Task (sec)	Performance Index (% Accuracy: Response Time)
0 mg	40.8 ± 1.8	44.3 ± 3.2	23.8 ± 2.4	54.5 ± 2.2	9.7 ± 1.8	0.42 ± 0.4
75 mg	42.5 ± 1.4	42.6 ± 2.7	26.9 ± 2.2	55.0 ± 2.7	7.3 ± 0.6	0.92 ± 0.9 *
150 mg	42.5 ± 1.8	45.3 ± 3.2	24.8 ± 2.2	55.9 ± 3.2	8.8 ± 1.4	0.42 ± 0.4
300 mg	42.8 ±1.7	45.7 ± 3.1	26.5 ± 2.2	55.7 ± 1.7	6.7 ± 0.4	0.97 ± 0.8 *

## Abbreviations

The following abbreviations are used in this manuscript:

3MS	Modified Mini Mental State Examination
AGEs	Advanced glycation end products
AUC	Area under the curve
BMI	Body mass index
BP	Blood pressure
CVR	Cerebrovascular responsiveness
ER	Estrogen receptors
FMD	Flow-mediated dilatation
GI	Glycemic index
MCA	Middle cerebral artery
NO	Nitric oxide
SS3	Serial Subtraction 3
T2DM	Type 2 diabetes mellitus
TCD	Transcranial Doppler
TMT	Trail Making Task
